# Properties of Self-Compacting Concrete Produced with Optimized Volumes of Calcined Clay and Rice Husk Ash—Emphasis on Rheology, Flowability Retention and Durability

**DOI:** 10.3390/ma16165513

**Published:** 2023-08-08

**Authors:** Abubakar Muhammad, Karl-Christian Thienel

**Affiliations:** Institut für Werkstoffe des Bauwesens, Universität der Bundeswehr München, 85577 Neubiberg, Germany; christian.thienel@unibw.de

**Keywords:** self-compacting concrete, calcined clay, rice husk ash, reactivity, flowability retention, rheology, flow resistance, durability

## Abstract

The durability of concrete requires a dense microstructure which can be achieved by using self-compacting concrete (SCC). Both calcined clay (CC) and rice husk ash (RHA) are promising supplementary cementitious materials (SCMs) that can partially replace cement, but their use in SCC is critical due to their higher water demand (WD) and specific surface area (SSA) compared to cement. The effect of partial substitution of cement at 20 vol-% with binary and ternary blends of CC and RHA on flowability retention and durability of SCC was investigated. The empirical method of SCC design was adopted considering the physical properties of both CC and RHA. The deformability of the SCC was evaluated using the slump flow and J-ring tests. The T_500_ time and the V-funnel test were used to assess the viscosity of the SCC. The flowability retention was monitored by the plunger method, and flow resistance was determined based on the rheological measurements of SCC. The evolution of the hydrate phases of the binder in SCC was determined by thermogravimetric analysis, while the durability was evaluated by a rapid chloride migration test. Cement partial replacement with 20 vol-% CC has no significant effect on fresh SCC, flowability retention, compressive strength and durability properties. On the other hand, 20 vol-% RHA requires a higher dosage of SP to achieve self-compactability and increase the viscosity of SCC. Its flowability retention is only up to 30 min after mixing and exhibited higher flow resistance. It consumes more calcium hydroxide (CH) and improves the compressive strength and chloride resistance of SCC. The ternary blending with CC and RHA yielded better fresh SCC properties compared to the binary blend with RHA, while an improved chloride penetration resistance could be achieved compared to the binary CC blend.

## 1. Introduction

For decades, concrete has provided the infrastructure that was synonymous with economic growth and development, partly because of its peculiar mechanical and durability characteristics, ease of production and placement, and the wide availability of its constituents [1]. After hardening, concrete becomes a robust stone-like material capable of withstanding the effects of deteriorative mechanisms such as the ingress of harmful chemicals, freeze–thaw cycle, rain storms or other harsh environmental conditions [2,3]. Therefore, concrete requires a dense microstructure that can provide adequate cover to the embedded reinforcement bars and resist the ingress of harmful substances, which could jeopardize the concrete itself. One possible way to ensure better compaction of the concrete and for filling all the corners of formwork and embedding the steel reinforcements is to adopt a self-compacting method of concrete production [4,5].

A self-compacting placement method ensures homogeneous deformation of the fresh concrete, under the influence of gravity, to fill all the gaps and corners of the formwork and embed the reinforcement bars without the need for external vibration or compaction, thereby ensuring a homogeneous and dense hardened concrete microstructure with excellent strength and durability [6]. The flowability of the SCC is achieved by proper optimization of the volumetric water-to-powder ratio (V_w_/V_p_) and the use high range water-reducing admixtures [7], while the homogeneity of the powder type of SCC is achieved by an optimal proportioning of fine and coarse particles of the concrete constituents [6]. In this regard, the proportion of coarse aggregate (CA) is reduced while the volume of mortar in the system is increased. Consequently, the quantity of the powder required to achieve optimized fresh SCC properties (powder-type) is greater than that of the conventional vibrated concrete [8], making the concrete more expensive and less environmentally friendly.

One potential way to make SCC cost-effective and reduce the effects of excessive use of cement per m^3^ of SCC—such as shrinkage and high CO_2_ emission—is to reduce the amount of cement per m^3^ of SCC. This can be achieved by partially replacing cement with filler and/or supplementary cementitious materials (SCMs). The most commonly used natural filler in SCC is limestone powder (LP), which can replace up to 35 wt.% of cement clinker [9], thereby reducing cement interparticle friction and decreasing the flow resistance of the SCC [10,11] and promoting the reaction of C_3_S and the formation of AFm phases (hemi/monocarboaluminate) [12,13]. SCMs such as fly ash, silica fume, metakaolin, rice husk ash (RHA) and others can also be used as partial cement substitutes in SCC. Binary and ternary cement partial replacement with fly ash up to 30 wt.% improves the durability of SCC and reduces its drying shrinkage [14,15]. Higher cement partial replacement by fly ash will soon no longer be possible due to the decline in coal combustion enforced to limit global CO_2_ release. Similarly, silica fume at 10 wt.% cement partial substitute improves the compressive strength and durability of SCC [16,17], but its use at higher dosage is also limited due to its cost, high water demand and portlandite consumption. 

CC are anhydrous aluminosilicate pozzolanic materials that can partially replace cement at high proportions and improve the strength and durability of concrete. Metakaolin is the most reactive CC in concrete and mortar due to its higher early strength compared to other CCs [18,19,20]. However, due to its frequent use in other competing industries, metakaolin is an expensive material and less available compared to the other 2:1 CCs. Although the relevance of other common CCs for conventional vibrated concrete has been investigated in recent years [21,22], their application should also be extended to SCC. RHA, on the other hand, is a silica-rich SCM that can be used as a viscosity modifying agent or as a partial replacement to cement in SCC [20,23,24,25]. Its specific SSA and WD exceed those of cement and metakaolin [20,23]. Therefore, similar to silica fume, its use in SCC is limited due to its effect on the deformability characteristics of SCC. It is obvious that both metakaolin and RHA at a certain cement replacement ratio densified the concrete microstructure, improving its chloride penetration resistance, increasing compressive strength and decreasing drying shrinkage tendency, but both are known for their high SSA and high WD, both significantly exceeding those of cement [20]. Consequently, the flow resistance of SCC is increased when cement is replaced with metakaolin and RHA, as the flow resistance of the blended cement increases due to the increase in the WD of the system [26].

SCC usually differs from conventional vibrated concrete only in its fresh properties. Its deformability characteristics and flowability retention are important attributes that determine its application. Previous investigations have established the potential use of binary and ternary blends of CC and RHA as partial replacements to cement up to 30 wt.% [27,28,29,30,31]; most of these investigations assessed the influence of these materials on the deformability of SCC only up to 30 min after production. Flowability retention of SCC is important, especially when considering applications requiring extended fresh properties retention. For instance, in the precast concrete plant, SCC can be cast into forms about 10 to 30 min after mixing, and the flowability retention is not as important as in the ready-mix concrete, which often requires 30 to 120 min after mixing [32]. The major concern at this point is the peculiar physical properties of CC and RHA, both of which have higher WD and SSA greater than cement, although RHA is more critical in this context because it can absorb the mixing water, causing the SCC to lose its stability quickly. Although maintaining the flowability of the SCC over an extended period of time is not readily achievable, it is important that the ready-mixed SCC still exhibit the required fresh SCC properties at the time of placement. None of the previous studies have reported the effects of CC and RHA on the flowability retention of SCC. 

The deformability characteristics of SCC after mixing can be assessed by measuring the yield value (slump flow), which defines the flowability of the SCC, and by checking the deformation rate by V-funnel measurement as an indication of viscosity [33,34]. The SCC can then be finally analyzed for its blocking and segregation tendency [35]. These properties are usually measured within 30 min of mixing and are not indicative of the flowability retention of the SCC. The flowability retention of SCC can be assessed by determining the immersion depth of the plunger in SCC in a cylindrical mould, as developed by FIZ (Research Institute of the Cement Industry) Düsseldorf [36], or by comparing the flow resistance of the SCC over time, calculated from the curves of shear stress versus shear rate from the rheological measurements.

## 2. Research Gap

Previous studies have investigated the suitability of a multi-blend of CC and RHA for the production of the SCC, as reviewed by [20]. Most researchers focused on the use of metakaolin as CC, which is an expensive material due to its frequent use in other competing industries and is less available compared to low-kaolinitic CC [23,24]. There is a lack of knowledge when it comes to the use of low-kaolinitic CC as a sole SCM or in combination with RHA as a partial replacement for cement in SCC, as pointed out by [20]. More so, previous studies have investigated the effects of blends of metakaolin and RHA on the fresh properties of SCC up to 30 min after production. However, the effects of these materials on the flowability retention and rheological properties of SCC in terms of yield stress, plastic viscosity, shear thickening behavior, plastic shrinkage and drying shrinkage have not been reported. Therefore, current efforts are aimed at optimizing the SCC mix design using a multi-blend of low-kaolinitic CC and RHA as a partial replacement for cement. Other properties to be investigated include the rheology, flowability retention, evolution of hydrate phases of the binder, plastic and drying shrinkage, compressive strength, and rapid chloride migration. The outcome of the study shows the effects of CC and RHA on the flowability retention and durability of SCC and the potential use of these materials to replace a high proportion of Portland limestone cement (PLC) in SCC, leading to a reduction in production costs and environmental impact.

## 3. Materials and Methods

### 3.1. Research Materials

Self-compacting paste, mortar and concrete are produced using CEM I 42.5 R, conforming to DIN EN 197-1 [9]. The cement (OPC) is partially replaced by 15 vol-% LP to form a Portland limestone cement (PLC). The mineralogical compositions of OPC (according to manufacturer’s data) and LP (determined by X-ray diffraction) are provided in Table 1. 

The PLC is partially substituted with the binary and ternary blends of CC and RHA up to 40 vol-% to determine the influence of CC and RHA on the rheological properties of SCC. The raw material for CC is an Amaltheen clay from southern Germany, which was calcined on an industrial scale at 750 °C and ground with an industrial roller mill to the fineness described in Table 2. The CC contained (wt-%) 60.8 amorphous phase, 2.2 muscovite, 16.2 quartz, 4.6 illite, 0.6 calcite and 1.6 sulfates mineralogical phases as characterized previously by [37]. For the RHA, rice husks from the vicinity of Zaria in Kaduna State, Nigeria, were calcined at 650 °C for 2 h in an electric furnace and ground with a laboratory scale mill to obtain the RHA. It contained 96.8% wt.% amorphous silica as the mineral phase, measured using X-ray diffraction with an internal standard. Table 2 shows the physical properties of the research binders. The particle shape of CC and RHA was examined using Evo LS 15 (Zeiss, Jena, Germany) scanning electron microscope (SEM). Both CC and RHA were scattered on a thin adhesive layer and coated with gold before SEM images were taken. 

**Figure 1:**
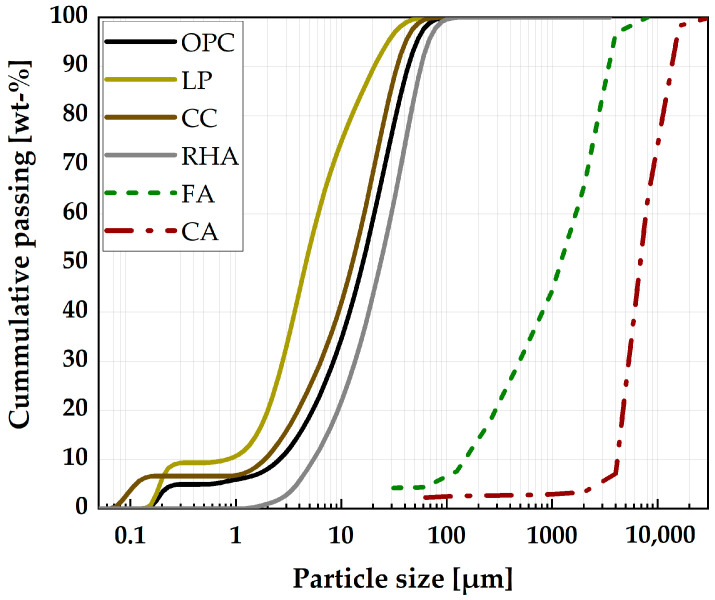
Particle size distribution of the research materials.

The fluidity of the SCC is controlled with the use of superplasticizer (SP), which has a total solid content of 38.6 wt.%, 1390 micromoles/g anionic charge density, 25,992 g/mol molecular weight and a side chain length of n_EO_ = 31 according to the supplier’s information. To complete the SCC mix design, sand with a grading of 0 to 4 mm, a particle density of 2.87 g/cm^3^ and having 7.6 wt.% passing sieves 0.125 mm was used as fine aggregate (FA). While round gravels with a grading of 4 to 16 mm, a particle density of 2.68 g/cm^3^ and 2.7 wt.% passing sieves 0.125 was used as CA. Figure 1 shows the particle size distribution of the research materials.

### 3.2. Methods

#### 3.2.1. Self-Compacting Paste, Mortar and Concrete Mix Design

The powder type of SCC used for the study was designed using a stepwise empirical method. This implies designing the paste phase of the concrete first. The amount of water confined by the binder is determined using the spread flow method, according to [41]. At a constant volume of the powder, the volumetric water-to-powder ratio (V_w_/V_p_) is varied between 1 and 2 at 0.1 intervals. Four points were used for each binder system, and the relative spread is determined using Equation (1). The V_w_/V_p_ is plotted against the relative spread, and the amount of water confined by individual binder system (V_wΓo_) is determined by extrapolating the points to the vertical axis [5].

Γ_RS_ = [d/d_o_]^2^ − 1 (1)

where Γ_RS_ = relative spread area, d = (d_1_ + d_2_)/2, d_1_ = largest flow diameter and d_2_ = diameter perpendicular to d_1_, and d_o_ = diameter of mini-slump cone, 100 mm.

The influence of CC and RHA on the amount of water confined by the PLC was determined and was used as the basis for establishing the V_w_/V_p_ of the self-compacting paste systems (SCP) using Equation (2).

V_w_/V_p_ = [0.8 to 0.9] ∙ V_wΓo_ (2)

where V_wΓo_ = volume of water confined by binder in relation to its solid volume.

The amount of the SP required to deform the SCP systems was determined as the dosage of SP at which a particular binder system attained a flow diameter of 240 mm ± 20 mm—a flow diameter at which the SCP achieved deformability without segregation based on visual inspection. Self-compacting mortar (SC-M) design was achieved by considering the average V_w_/V_p_ required to deform SCP, and the volume of the FA (V_s_) is fixed at 44 vol-% of the SC-M volume, according to [41]. The amount of SP is adjusted until the individual SC-M system’s self-compactability is attained. The deformability of the SC-M systems was assessed by measuring the slump flow using the mini-slump cone [5,41], and the range of 240 mm to 260 mm was used as a yardstick for indicating the deformability limit at which self-compactability is achieved. The viscosity of SC-M was monitored by measuring the mini V-funnel time [42], while visual inspection was used to evaluate the segregation resistance of the SC-M. To complete the SCC mix design, the volume of the CA was adjusted by intense testing together with varying the dosages of the SP until it was kept at 33% of the total SCC volume. The air content was also fixed at 2 vol.% of total SCC volume, according to [41].

#### 3.2.2. Batching and Mixing of Self-Compacting Paste, Mortar and Concrete Constituents

SCP mixes were produced according to DIN EN 196-1 [43]. The binder, mixing water and SP were added together and mixed for 4 min in a mortar mixer produced by Bluhm and Feuerherdt GmbH, Berlin, Germany. SC-M was produced by mixing the constituents for 8 min in the same mixer described above. The binder materials, FA and two-thirds of the mixing water were mixed for 2 min, followed by a break of 1 min to enable the addition of the remaining water and the SP. The entire mixture was then mixed for an additional 5 min. The mixing sequence of SCC started with mixing the binder, FA and CA for 1 min in a UEZ ZM 50 concrete mixer (UEZ-MISCHTECHNIK), with a capacity of 60 L and a mixing speed of 48 rpm. Two-thirds of the mixing water was then added while the mixer was running and mixed for 2 min. The SP and the remaining mixing water were then added, and the whole constituents mix for another 7 min.

#### 3.2.3. Fresh SCC Properties and Segregation Resistance

The deformability of the SCC in absence of obstacles was evaluated using the slump flow according to [33], while the J-ring test was employed to assess the passing ability of the SCC through an obstacle according to [35]. The viscosity of the SCC systems was assessed using both the T_500_ time and the V-funnel test, according to [34]. The stability of the SCC was evaluated in two ways. First, using the sieve stability assessment method according to [44] and the washing test according to [45] where the fresh SCC was poured into a plastic cylinder 150 × 500 mm (with a solid base and cut at third points up to half of the circumference to accommodate metal dividers). The cut points for receiving the metal dividers were sealed with adhesive tape before pouring the SCC. Immediately after mixing, the SCC was poured into the cylinder and stored vibration-free for 30 min until the time of testing. After 30 min, the adhesive tape is removed, and the metal dividers are inserted. The SCC is then poured into vessels in segments and washed through 4 mm aperture sieve. The sieve residue was dried in a ventilated oven at 105 °C and weighed.

#### 3.2.4. Rheology and Flowability Retention Assessment

The uniform distribution of the CA in the SCC mixtures is monitored over time using a plunger method [36]. SCC is poured into a hollow cylinder with an internal diameter of 150 mm and a height of 600 mm. A 900 g steel plunger with a diameter of 14 mm and a height of 750 mm is then guided through the guide tube (with 3 openings) to the surface of the SCC and released to penetrate into the SCC. The depth of immersion of the steel plunger is measured, and the sedimentation height (h_s_) is calculated as the difference between the steel plunger height (h_0_) and the immersion depth (h_T_) in mm. The steel plunger and the guide tube are withdrawn, cleaned and dried for the next measurement. Previous immersion holes were avoided by changing the position of the guide tube after the whole available guide holes had been exhausted.

Rheology of the SCC in terms of the shear stress (τ), plastic viscosity (µ) and flow resistance were determined using a rotational rheometer, viskomat XL (Schleibinger Geräte, Buchbach, Germany), with a vane probe testing paddle at a constant temperature of 20 °C. The measurement started with the maximum rotational speed of 12 rpm for 80 s and decreased in 14 steps of 20 s each until the end of the measurement [46]. The recorded torque was used to estimate the yield stress (τ_0_) and plastic viscosity (η) of the SCC according to the Bingham model [47,48] using Equation (3). The flowability retention of the SCC is monitored by calculating flow resistance of the SCC mixtures over time (area under curve) obtained by plotting the torque against the velocity as described previously by [26].

(3) T=G+N · H

where *T* = torque ≈ τ; *G* = y-intercept of the flow curve ≈ τ_0_; *N* = velocity ≈ *γ*˙; and *H* = the slope of the curve ≈ η.

#### 3.2.5. Portlandite Consumption, Plastic and Drying Shrinkage of Self-Compacting Paste, and Mortar

The plastic shrinkage of SC-M at an early age was measured contactless using a shrinkage cone (Schleibinger Testing Systems, Buchbach, Germany), according to [49]. The measurement started 15 min after water addition and lasted for up to 48 h. Polypropylene foil was used to prevent the friction effect between the fresh SCC surface and the shrinkage cone. The SC-M drying shrinkage was measured on 40 × 40 × 160 mm^3^ prisms, according to [50]. The portlandite (CH) consumption by the CC and RHA and hydrate phases formed at 2, 7 and 28 days of curing were investigated by thermogravimetric measurements (TG) conducted in Netzsch STA 449 F3 Jupiter apparatus. The constituents of the SCP were mixed according to [43], poured into 40 × 40 × 160 mm^3^ steel molds and stored moist for 48 h, and then cured under water until testing. At the time of testing, a representative portion of the sample is manually chipped out from the inner part of the prisms and crushed to <1 mm size using the laboratory pestle and mortar. Hydration of the pulverized binder was stopped by solvent exchange, as described in [51]. Approximately 300 mg of the pulverized binder was placed in alumina crucibles and heated to 1000 °C at a heating rate of 2 °C/min under a nitrogen atmosphere. The CH content of the SCP was quantified by the tangent method from the weight loss between 400 °C and 490 °C.

#### 3.2.6. Compressive Strength and Chloride Migration Resistance of SCC

For the compressive strength measurement, SCC was cast in 150 × 150 × 150 mm^3^ steel molds and stored moist for 48 h and then cured underwater until the test date. The measurement was conducted according to [52] at 2, 7 and 28 days of curing on the Form + Test Prüfsysteme Alpha 1–3000 strength testing equipment with an increasing uniform loading rate of 2400 ± 200 N/s until failure. The influence of CC and RHA on chloride resistance of SCC was investigated using a rapid chloride migration by applying an electric field axially to the test specimen to accelerate the chloride penetration, according to [53]. The depth of chloride penetration was determined on 100 × 50 mm^3^ cores drilled and subsequently sliced from 150 × 150 × 150 mm^3^ SCC specimens at day 28 of curing.

## 4. Results and Discussion

### 4.1. Optimization of SCC Mix Design with the Blend of CC and RHA

**Figure 2:**
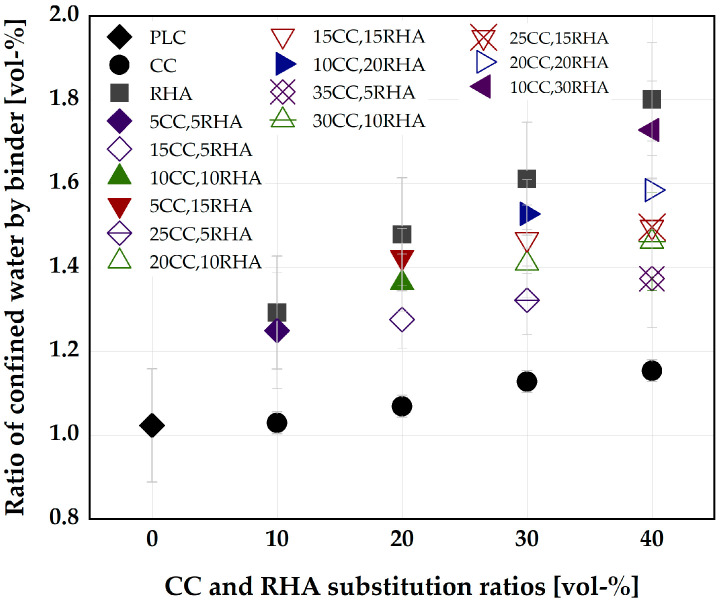
Influence of CC and RHA on the ratio of confined water by PLC.

To optimize the blend of CC and RHA as a partial replacement for PLC in SCC, the influence of CC and RHA on the amount of water confined by the binder was first considered. PLC without CC and RHA partial replacement confined approximately a volume of water equal to the volume of solid. A substitution by 10 vol-% CC showed no significant effect on the volume of water confined and increased to 11 vol-% of water at a substitution ratio of 40 vol-%. RHA at 10 vol-% PLC substitution confined 20 vol-% additional water compared to the PLC, and the volume of water confined increased to 43 vol-% at an RHA substitution ratio of 40 vol-%. Figure 2 shows the ratio of confined water (vol-%) by PLC, CC, RHA and the ternary blends of CC and RHA.

**Figure 3:**
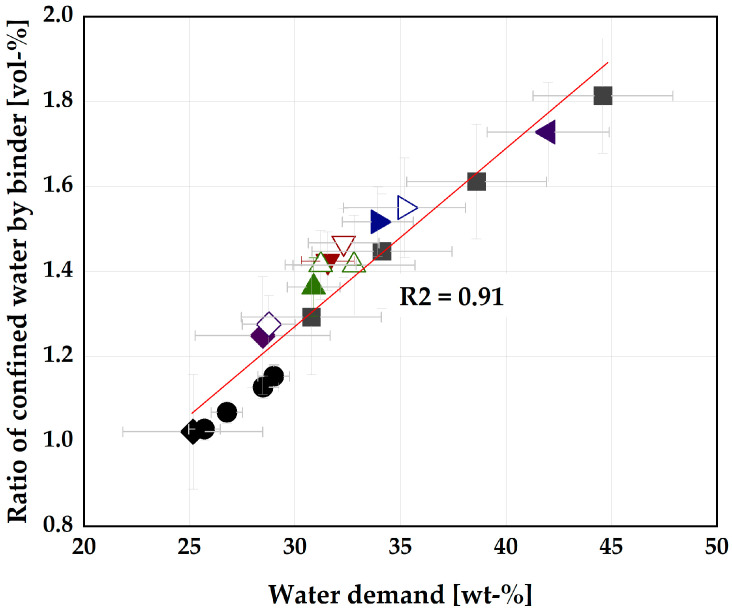
Relationship between the water demand and the ratio of water confined by the binder; the same legend used in Figure 2 is applicable here.

However, the ternary blends of CC and RHA yielded no reduction in the V_wΓo_ due to the high-water demand of RHA. For instance, the V_wΓo_ for 40 vol-% CC replacement is 1.15 and increased drastically to 1.37 when 5 vol-% of CC is replaced by RHA in the blend (35CC, 5RHA). Figure 3 shows the relationship between the water demand and the ratio of confined water by the binder systems. The ternary blends of CC and RHA appeared above the line of best fit, while the binary blends appeared on the line or below.

**Figure 4:**
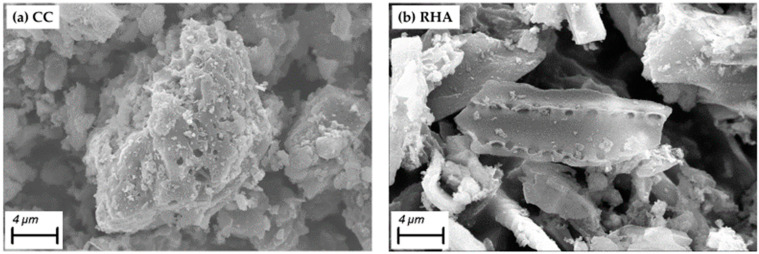
Particle morphology of (**a**) heterogeneous CC and (**b**) granular irregular RHA.

A possible explanation for this behavior of the ternary blends is the dominating influence of RHA on the properties of the blended binder. Even a small amount of RHA in the ternary blend leads to an increase in both the water demand and the amount of water trapped by the binder compared to the binary CC mixes. It could also be due to improper mixing of the two materials since CC has a heterogeneous surface morphology, while RHA contains an irregular, granular surface of isolated plate morphology, as shown in Figure 4, so the presence of CC in the mixture does not have a significant effect on reducing the water demand of ternary mixtures.

The design of SCC starts with the determination of the V_w_/V_p_ required to impart on-paste self-compactability. This V_w_/V_p_ is calculated from the volume of water confined by the individual binder system, and the average of these values for the individual binder systems is shown in Table 3. PLC requires an average of 0.87 V_w_/V_p_ to achieve self-compactability. Partial substitution of CC up to 20 vol-% has a minor effect on this value; CC partial substitution beyond 20 vol-% requires an adjustment of this value or an increase of the SP dosage to achieve a similar deformability class as the PLC system, as a previous investigation by [54] achieved self-compactability using the same V_w_/V_p_ and increasing SP dosage, with up to 40 vol-% CC in SC-M. On the other hand, the partial substitution of PLC with RHA requires a significant increase of V_w_/V_p_ to achieve self-compactability. At 10 vol-% RHA partial replacement, V_w_/V_p_ already increased to 1.1, which is 21% higher than the value required for PLC. At 40 vol-% RHA partial replacement, the V_w_/V_p_ increased by 42%. In this case, the SP adjustment is not sufficient to achieve the required degree of deformability because of the higher water demand and specific surface area of the RHA compared to PLC. Therefore, urgent adjustment of the V_w_/V_p_ of the RHA and the ternary blend of CC and RHA systems is required to achieve a deformability characteristic similar to that of PLC. 

The SCP design in this section will later be used as a binder in SC-M and SCC. Therefore, the selection of the V_w_/V_p_ will be carried out considering the intended final use of SCC. The following outlook is considered to justify the selection of V_w_/V_p_ for the SCP design. In practice, the water-to-binder (w/b) and the strength of the binder determined the strength and durability class of the concrete and, thus, its application as provided in the concrete design standards [55,56]. To achieve SCC with PLC, a V_w_/V_p_ = 0.87 is required, corresponding to a w/b = 0.29; for CC replacement, up to 40 vol-%, a w/b = 0.32 is required. For 10 vol-% RHA, w/p = 0.36 is required, increasing to 0.42, 0.45 and 0.50 for 20, 30 and 40 vol-% partial replacement, respectively. When using a macro-mesoporous RHA as a partial replacement to cement in SC-M, Le et al. [57] achieved self-compactability using a w/b = 0.26 with up to 20 wt.% RHA as a partial replacement for cement, and despite increasing dosages of SP, RHA increased the viscosity of the SC-M. It should be noted at this point that the deformability of the SC-M does not necessarily indicate the deformability of SCC because SCC contains a large volume of coarser aggregate in addition to SC-M, which has a significant impact on its deformability characteristics. By ternary blending residual RHA and LP as partial replacement to OPC, Sua-iam et al. [58] achieved self-compactability using a low w/b = 0.28 and the same dosage of high-range water reducers with RHA substation up to 20 wt.%, but the viscosity of 20 wt.% RHA binary blended SCC was 63% higher than the control (viscosity = V-funnel time). Therefore, a lower w/b value (≤0.3) does not provide the degree of deformability required when ≥ 20 vol-% RHA is used as a partial replacement for cement. The same deformability characteristic as the reference systems with higher RHA replacement ratios (≥20 wt.%) were achieved by increasing the w/b to 0.44 and above [27,30,59]; similar values of w/b are assumed for this investigation as the PLC partial replacement level is kept at ≥20 wt.%. Another factor that governs the selection of the w/b is the expected performance of the SCC after hardening. For chloride exposure under alternating wet and dry conditions, the maximum allowable w/b is 0.45 [55].

**Figure 5:**
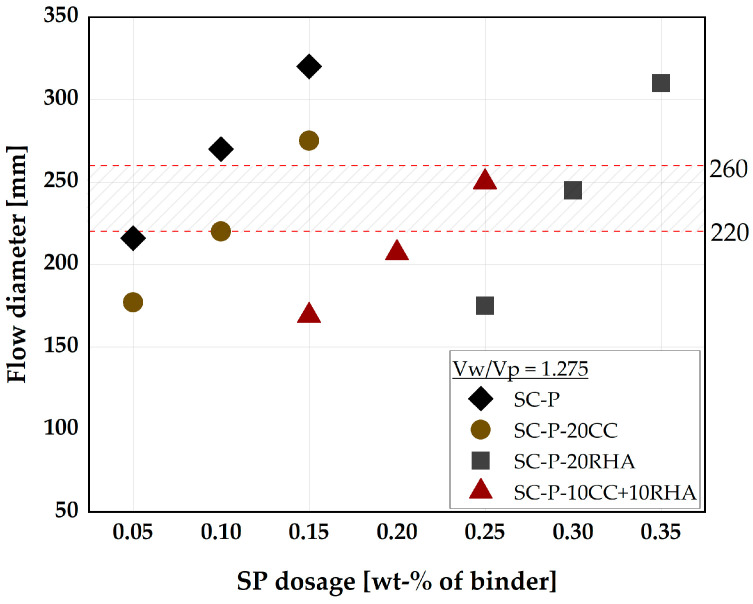
SP dosages required to deform SCP systems.

Partial replacement of PLC with CC, even up to 40 vol-%, yields the required deformability characteristics and has wide applicability in different durability exposure classes, as previously reported by [54,60], while providing significant savings in production cost and reducing CO_2_ emission. Higher PLC partial substitution with RHA up to 40 vol-% is also possible using a higher w/b of ≥0.5; this concrete could also have a wide range of applications, for instance, in exposure classes X0, XC, and some classes of XD and XS exposure, according to [55]. Considering that RHA poses a challenge to deformability at higher PLC partial substitution due to its high WD, this study will limit the RHA replacement ratio to 20 vol-%. For comparison, the binary blend with CC will also be kept at 20 vol-%, and the ternary blend will use 10 vol-%CC + 10 vol-%RHA. Therefore, V_w_/V_p_ = 1.275 required to impart self-compactability to the 20 vol-% RHA binder system is considered as the V_w_/V_p_ in the following sections of this paper and is used to establish the SP dosages required to deform the SCP systems, as depicted in Figure 5.

Based on the visual inspection, SCP achieved deformability without segregation at a flow diameter of 220 to 260 mm. Therefore, the dosage of SP is established, which is necessary for the individual binder systems to achieve a flow diameter of 240 mm ± 20 without segregation. The latter is assessed visually. PLC binder system (SC-P) requires an SP dosage of between 0.05 to 0.1 wt.% to achieve self-compactability. SC-P-20CC demands a slightly higher SP dosage than SC-P, while for the SC-P-20RHA system, the SP dosage already increases drastically to 0.3 wt.%, although this is reduced to 0.25 with the SC-P-10CC+10RHA blend. The increase in the SP demand is due to the higher WD of RHA, which continues to trap water in its structure and requires more SP to achieve the required degree of deformability. The final SCP mix design is shown in Table 4 and was used as the basis for SC-M design and to measure the influence of CC and RHA on the formation of hydrate phases over the period of 28 days of curing.

**Figure 6:**
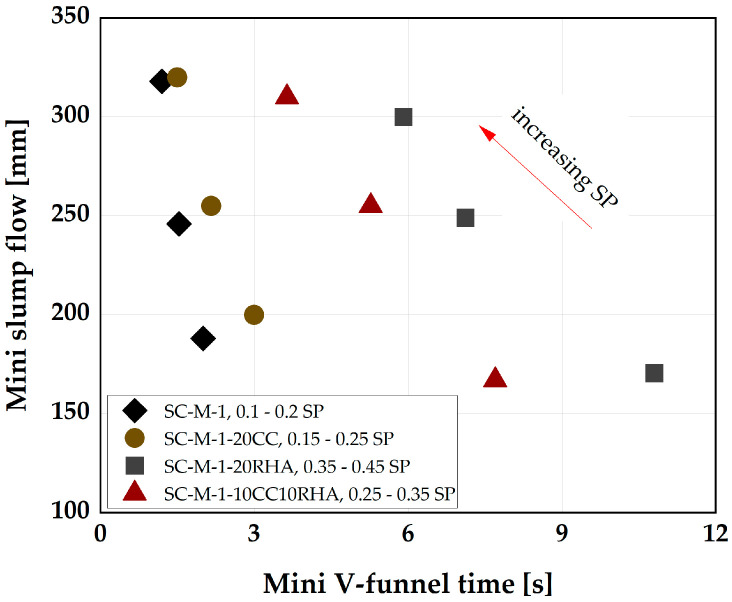
Influence of CC, RHA and SP dosage on the deformability characteristics of SC-M.

The SCP designed above were used in the next step as media to deform the SC-M and bind the fine aggregate. The SC-M mix designs are achieved by fixing V_s_ at 44 vol-% of the total SC-M volume, based on the recommendation of [41]. SP dosages are adjusted to achieve the required degree of deformability. Figure 6 shows the deformability and the rate of deformability of the individual SC-M systems. 

With a slight adjustment of the SP dosage, SC-M-1-20CC achieved a similar deformability to SC-M-1, SC-M-1-20RHA, and the blend SC-M-1-10CC+10RHA could achieve a similar deformability class to SC-M-1 but with an increased viscosity and higher SP dosages. For deformability assessment, EFNARC’s [41] guidelines were adopted. A range of 240 mm to 260 mm flow diameter and a viscosity class (V-funnel time) of 7 s to 11 s were used. Only SC-M-20RHA falls under these limits. The remaining mixtures exhibited a higher flow rate, indicating low viscosity. Therefore, mixtures that are stable (judged by visual assessment) between the flow diameter = 240 mm to 260 mm were considered for the production of the SC-M specimens. Table 5 provides the final SC-M mix designs, which were used to monitor the influence of CC and RHA on plastic and dry shrinkage strains.

**Figure 7:**
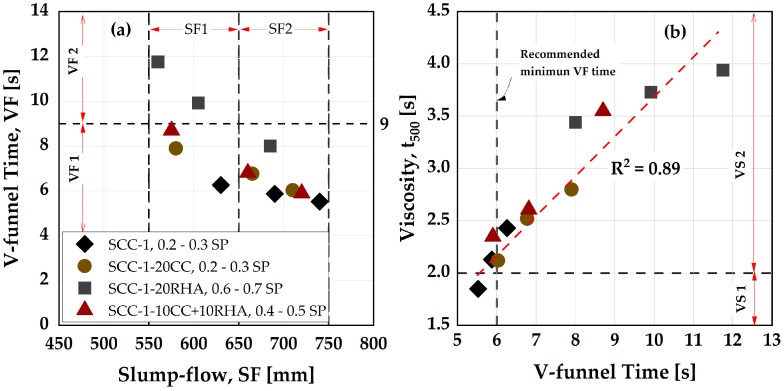
(**a**) Influence of CC, RHA and varying SP dosages on the deformability characteristics of SCC. (**b**) Relationship between the VF and t_500_. Explanations for the symbols are provided in (**a**).

The final SCC mix design is achieved with a fixed CA content of 33 vol-% and assuming an air content (V_a_) of 2 vol-% of the total SCC volume according to EFNARC [41], while the SC-M designed above complete the remaining SCC volume. The SP dosages were adjusted until an acceptable deformability was achieved, as shown in Figure 7a. The criteria for assessing the deformability of SCC in the absence of obstacles (filling ability) include the slump flow, V-funnel and t_500_ times as an indication of viscosity, while the use of J-ring can give an estimation of the deformability in the presence of an obstacle (passing ability). The combination of these assessments was used as the basis for selecting the appropriate mixes to produce the final SCC. By adjusting the SP dosages, SCC-1 achieved a deformability class SF2 and viscosity class VS1 and VF1, according to [56]. With a slight increase in the SP dosage, SCC-1-20CC achieved similar deformability to the SCC-1, with viscosity classes of VS2 and VF1. SCC-1-20RHA exhibited higher viscosity than SCC-1 and requires an increased SP dosage to attain deformability class SF2 and viscosity classes VS2 and VF1. The ternary blend, SCC-1-10CC+10RHA, exhibited a deformability and viscosity behavior somewhat in-between that of binary CC and RHA SCC. The increased viscosity with RHA substitution is due to the higher water demand of RHA compared to PLC and CC, as depicted in Table 2. A relationship between the V-funnel time (VF) and the t_500_ (VS) measured together with the slump flow is observed with a high correlation [61], as shown in Figure 7b.

**Figure 8:**
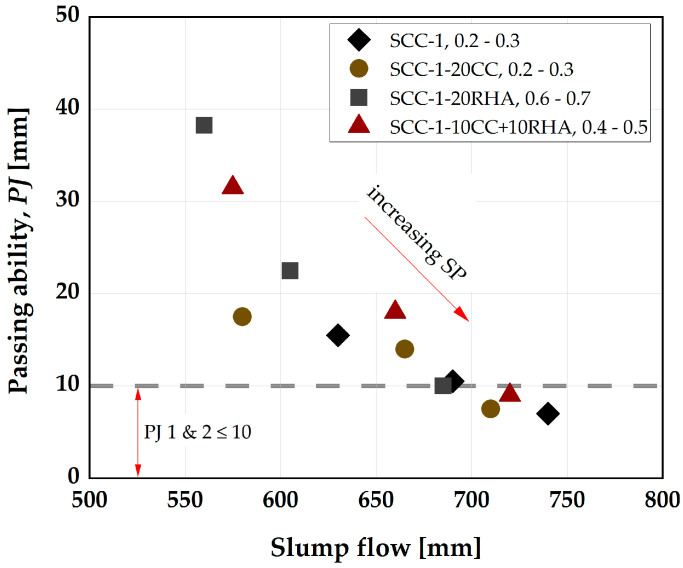
Influence of CC, RHA and varying SP dosage on the blocking tendency of SCC.

Similarly, by adjusting the SP dosages, the blocking tendency of the SCC decreases, and it is possible to bring all the SCC mixtures to a passing ability class PJ2, classified according to [56] (Figure 8).

The reference mixture (SCC-1) exhibits good passing ability using an SP dosage of 0.3 wt.%, but the viscosity (V-funnel time) falls below 6 s, the minimum recommended by [41] (Figure 7b). The tendency of segregation is high with this mixture, and therefore it was not considered for the production of the final SCC. The viscosity of the SCC depends largely on the w/b: The higher the w/b, the higher the rate at which the SCC will flow, as previous investigations obtained VF time ≤ 6 s using w/b ≥ 0.5 [4,62]. When the w/b is <0.4, SCC have a VF time ≥ 6 s [30,60]. SCC-1 with 0.25 SP dosage is at the limit for both the filling and passing ability values but is considered suitable for the final SCC mix design. The selection criterion for the final SCC mix design is therefore based on SF ≥ 650 mm, VF ≥ 6 s and PJ ≤ 10 mm and the proportions of the final SCC mix designations are presented in Table 6 and Table 7 and are used to determine the influence of CC and RHA on the segregation resistance, rheology, flowability retention, compressive strength and chloride migration resistance of SCC. 

The reference system (SCC-1) required a V_w_/V_p_ = 0.87, corresponding to w/b = 0.29, for self-compactibality, similar to that previously used by [54,58] to achieve high-strength SCC. Both CC and RHA have a WD greater than that of PLC. The WD of CC is one-third higher than that of PLC (see Table 2) but requires only an adjustment of SP to achieve a degree of deformability comparable to SCC-1, although with increased viscosity and SP demand as previously investigated by [29,54], when high volume of CC was used as partial replacement to cement. The increase in viscosity is not only due to the higher WD of the CC compared to PLC but also due to its different particle shape, as reported by [26,63], leading to an increased resistance to flow and hence higher SP demand to deform the SCC-1-20CC system. The use of RHA as a partial replacement for PLC in SCC is more critical due to its more than three times high WD (see Table 2). Unlike the SCC-1-20CC system, SP adjustment is not sufficient to deform SCC-1-20RHA and even the SCC-1-10CC+10RHA system. Therefore, V_w_/V_p_ adjustment is urgently needed to achieve self-compactability. When substituting a high proportion of PLC with RHA (20% and above), higher w/b, from 0.44 and above, will be required to properly deform the RHA-SCC system. This affects the concrete porosity and limits its application possibilities since the durability classes of concrete are determined on the basis of the w/p value, as already described in [55]. Therefore, this study limits the content of RHA to 20 vol-% and uses a Vw/Vp = 1.275 (0.42 w/b equivalent) for all PLC-, CC-, and RHA-SCC systems.

### 4.2. Influence of CC and RHA on the Segregation Resistance of SCC

**Figure 9:**
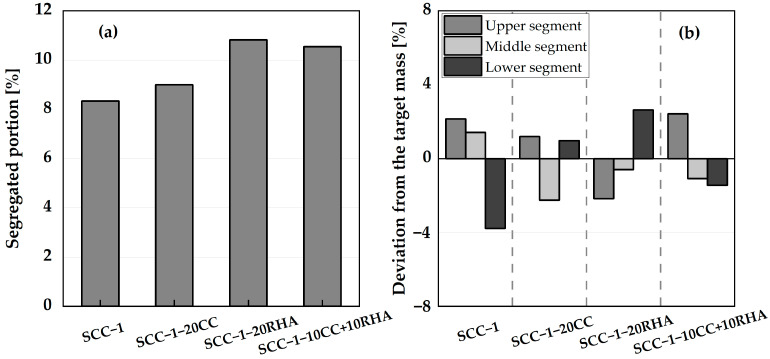
Sieve stability segregation resistance (**a**) and aggregate wash test (**b**) of SCC.

Both short-term and flowability retention assessments are conducted to establish the influence of CC and RHA on the stability of the SCC. For the short-term assessment of segregation resistance, sieve stability and washing tests are conducted after the fresh SCC has settled 30 min after mixing. Figure 9a shows the segregated portion of the SCC specimens determined by sieve stability. The segregated portion of all the SCC specimens is less than 11 wt.%, which is within the limit specified by [56], standard specification for SCC performance and conformity requirements. 

The proportions of the CA from the three segments of the cross-section of the fresh SCC evaluated by aggregate washing test are shown in Figure 9b. The mass deviation of the coarse aggregates from the three segments of all four test specimens is less than 4 wt.% and thus significantly below the limit (15 wt.%) specified in [45]. 

**Figure 10:**
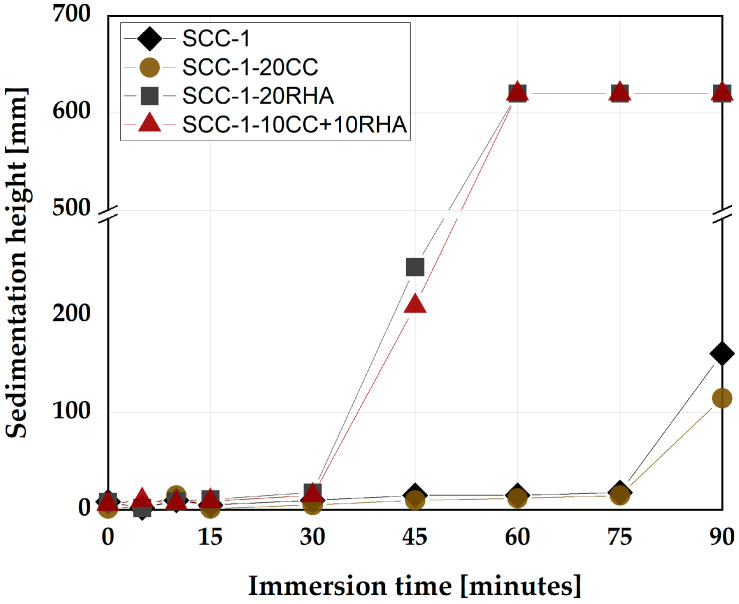
Flowability retention assessment of SCC.

The flowability retention of SCC specimens is monitored for the first 90 min by sedimentation analysis using a plunger method. SCC-1 and SCC-1-20CC remain stable until 75 min, indicated by the plunger almost reaching the bottom of the cylinder. At 90 min, the immersion depths decrease, indicating that the setting has started. SCC-1-20RHA and the ternary blend SCC-10CC+10RHA also showed similar sedimentation tendencies. In this case, the flowability retention lasts up to 30 min, beyond which the plunger only sinks halfway down the cylinder and at 60 min, penetration is virtually not possible due to the stiffening of the SCC surface (Figure 10).

**Figure 11:**
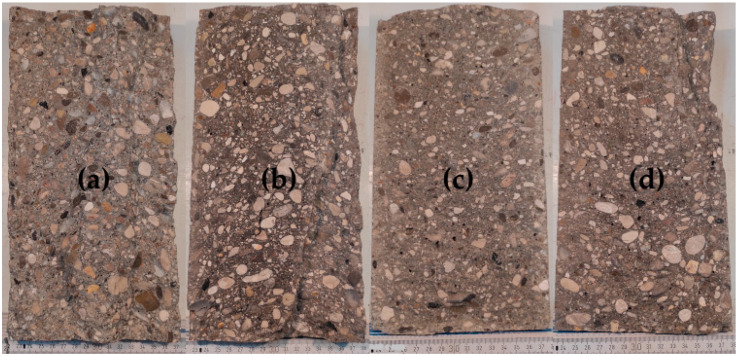
Cross-section of hardened SCC showing uniform distribution of FA and CA across (**a**) SCC-1, (**b**) SCC-1-20CC, (**c**) SCC-1-20RHA and (**d**) SCC-1-10CC+10RHA.

The short-term stability of all SCC mixes is within the specified limits by [45,56], and even SCC-1-20RHA retains its flowability up to 30 min after mixing. While for the flowability retention assessment, the use of RHA as a partial replacement for PLC is considered critical because the mixing water was absorbed after 30 min of mixing, making the mix stiffer and rapidly losing its self-compactability. The flowability retention assessment using the plunger method assumed the free sinking of the plunger down to the bottom of the concrete anytime it was guided until setting and hardening occur, which is about 90 min, depending on the type of cement used [36]. Excessive settlement of the CA during the early time of the SCC is considered to hinder the sinking of the plunger, signifying segregation had occurred. In this regard, SCC-1 and SCC-1-20CC behaved apparently stable and maintained their flowability until 75 min after mixing. For SCC-1-20RHA and SCC-1-10CC+10RHA, a rapid loss of flowability was observed after 30 min of mixing, which is attributed to the continued suction of the mixing water by RHA and not to the settlement of the CA since the hardened SCC with RHA as PLC partial replacement still exhibited a uniform distribution of the CA across its section, as shown in Figure 11c,d. 

Segregation resistance is an important attribute that determines the acceptability of SCC. It can be measured by sieve stability, which measures the bleeding of SCC after 30 min of resting. The SCC-1 system has a sieve stability value similar to what was obtained by [64], signifying that the result is similar to what is obtainable in the literature, and all the SCC systems meet the requirements of [56] for segregation resistance acceptance and can therefore be used in practice, especially when flowability retention beyond 30 min is not important (RHA-SCC mixes). The wash test was also used to measure the uniform distribution of the coarse aggregate over the fresh SCC section after 30 min of settlement, and all SCC specimens retained the coarse aggregate in suspension during the test period. The only flowability issue observed is the stiffening of the RHA-SCC systems, which is critical after 30 min of production due to the continuous suction of the mixing water by RHA, which may limit applications where longer retention of flowability is required.

### 4.3. Rheological and Flowability Retention of SCC

**Figure 12:**
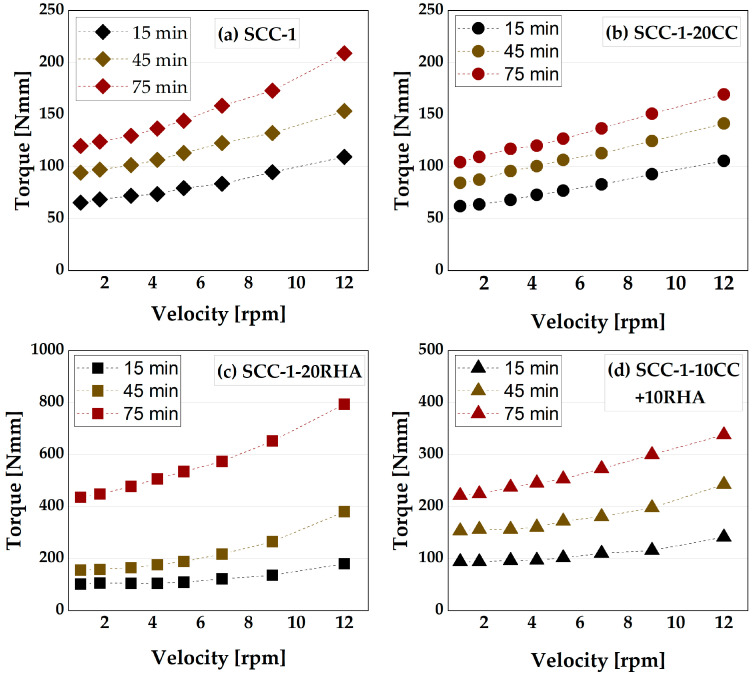
Influence CC and RHA on the time-dependent displacement of SCC-1 (**a**), SCC-1-20CC (**b**), SCC-1-20RHA (**c**) and SCC-1-10CC+10RHA (**d**).

The time-dependent rheological assessment of SCC begins with determining the influence of CC and RHA on the torque required to initiate and maintain displacement of SCC based on the applied shear rate measured with a viscometer, as depicted in Figure 12. At 15 min of testing, SCC-1 recorded a torque of 66 Nmm at a lower velocity of 1 rpm and 109 Nmm at the maximum applied velocity of 12 rpm. After 75 min of testing, the torque increased to 120 and 209 Nmm, respectively. SCC-1-20CC required lower torque to achieve and maintain displacement compared to SCC-1, both at lower and higher velocities and at all test times. Although CC has a higher water demand than PLC, the decreased torque could be due to the higher dosage of SP applied to deform the SCC-1-20CC system, despite both binder systems requiring almost similar V_w_/V_p_ to achieve self-compactability, as shown in Table 3. For the RHA binder system, on the other hand, at 15 min of testing, a torque of 102 Nmm was measured at lower velocity and 180 Nmm at higher velocity. These values increased significantly after 30 min of testing due to the stiffening of the SCC-1-20RHA mixture as a result of higher water demand of RHA (see Figure 10). The ternary blend with CC and RHA behaved somewhat in between the SCC-1-20CC and SCC-1-20RHA; the presence of the CC in the blend decreased the torque required to achieve displacement at 75 min of testing by 49 and 57% at lower and higher velocities, respectively, compared to RHA-SCC system.

**Figure 13:**
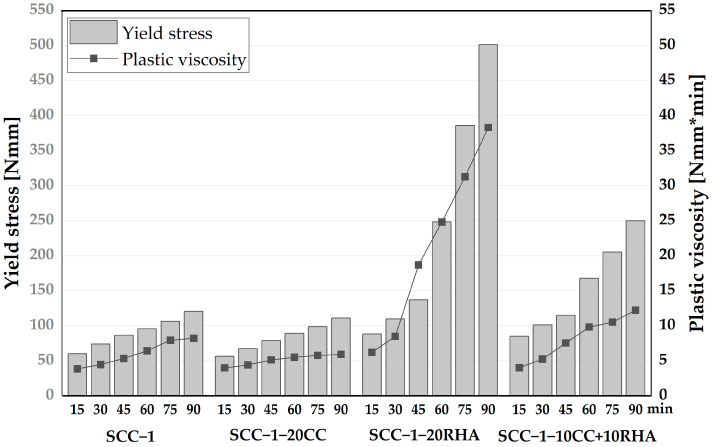
Time-dependent dynamic yield stress and viscosity values of SCC measured up to 90 min after water addition.

Establishing the dynamic yield stress of SCC is important to determine the extent of energy required to maintain SCC flow; this is important to ensure uniform deformability of SCC across formwork sections. The time-dependent yield stress and plastic viscosity of SCC were established from the measured torque values induced by the applied velocity using a Bingham model, as presented in Figure 13. SCC-1 has a yield stress of 60 Nmm and a plastic viscosity of 3.9 Nmm*min at 15 min of testing, which gradually increased to 120 Nmm and 8.2 Nmm*min, respectively, after 90 min of testing. SCC-1-20CC showed a similar increasing tendency in yield and viscosity values as SCC-1. The gradual increase of the yield stress and plastic viscosity could be due to the loss of water from the surfaces of SCC caused by evaporation or due to the chemical reaction between the binder and mixing water leading to the initial formation of ettringite, as explained previously in [65]. SCC-1-20RHA had a yield stress of 88.5 Nmm and a plastic viscosity of 6.2 at 15 min of testing, which are 47 and 59% higher than SCC-1, respectively. This yield stress and plastic viscosity values increased rapidly up to 90 min of testing, unlike the SCC-1 and SCC-1-20CC systems. The rapid increase of both yield stress and plastic viscosity values is due to the increased plastic stiffening caused by the continued absorption of the mixing water by the RHA particles. The ternary blend of CC and RHA had both lower dynamic yield, plastic viscosity values and a lower rate at which they increased compared to SCC-1-20RHA. In all cases, SCC mixtures containing RHA exhibited higher yield stress and plastic viscosity values due to their higher water demand. 

**Figure 14:**
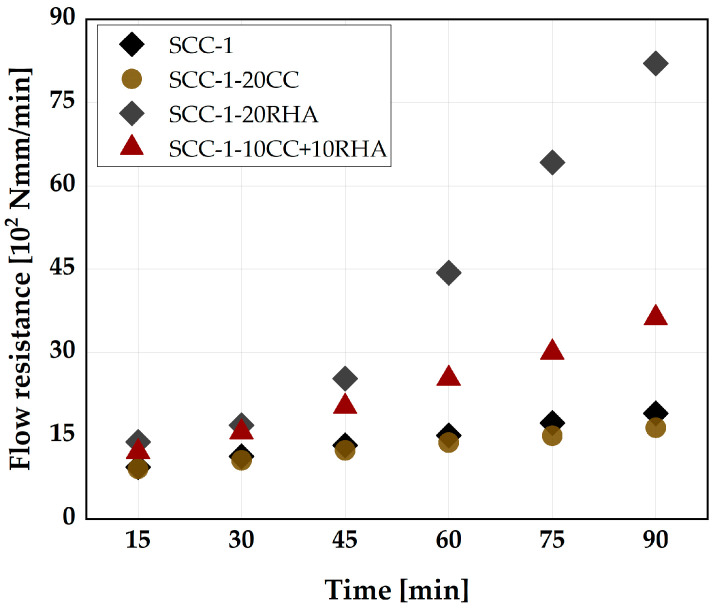
Effect of RHA and CC on the flow resistance of SCC.

The flowability retention of SCC is monitored by comparing the flow resistance of the SCC mixes over a period of 90 min. The flow resistance is determined as the area under the curve of the torque plotted against the velocity values from Figure 12. The flow resistance of SCC-1 measured 15 min after water addition is 933 Nmm/min and increases to 1900 Nmm/min after 90 min (Figure 14). SCC-1-20CC mixture exhibits similar flow resistance to SCC-1 up to 90 min of testing. SCC-1-20RHA, on the other hand, had a flow resistance value of 1388 Nmm/min at 15 min, which is 30% higher than SCC-1, and increased to 8205 Nmm/min at 90 min of testing. SCC-1-10CC+10RHA showed similar flow resistance to SCC-1-20RHA up to 30 min of testing, after which it increased less compared to SCC-1-20RHA. The decrease in flow resistance of SCC-1-10CC+10RHA is due to the presence of CC in the blend, which reduces the water demand and SSA of the system.

Generally, by increasing the SP dosage, PLC partial replacement with 20 vol-% CC has no effect on flowability retention of SCC up to 90 min and therefore, SCC-1-20CC can be used to produce both in situ, precast and ready-mix SCC. However, the use of 20 vol-% RHA and the ternary blend 10CC+10RHA developed high flow resistance and exhibited rapid loss of flowability after 30 min of mixing. Therefore, their flowability retention needs to be improved for applications beyond 30 min.

**Figure 15:**
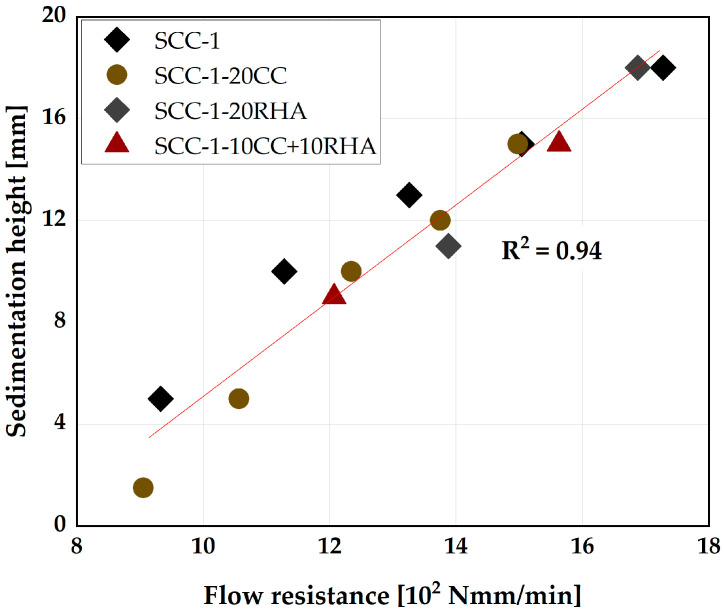
Relationship between the flow resistance and stability of SCC.

The relationship between the flow resistance measurement and the sedimentation analysis conducted by the plunger method is valid in the case of SCC-1 and SCC-1-20CC up to 75 min of testing, while for the SCC-1-20RHA and SCC-1-10CC+10RHA, the relationship is valid only up to 30 min of testing (Figure 15). The penetration of the plunger is not possible in the SCC mixes containing RHA after 45 min of testing due to the stiffening of the SCC surface, which hinders the plunger penetration. 

The differences between the two methods of flowability retention assessment could be explained by the mechanism of flowability loss due to structural build-up (thixotropy), which can easily be reversed during the viscometer rotation in the flow resistance assessment method, while for the sedimentation analysis, the plunger is only guided and allowed to penetrate the concrete on its own weight, the SCC remained undisturbed and structural build ups unreversed. 

### 4.4. Formation of Hydrate Phases from the Hardened SCP

**Figure 16:**
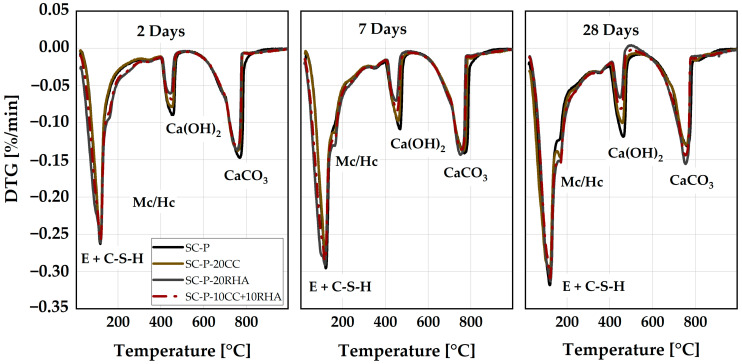
Differential thermal analysis of SCP specimens.

The formation of SCP hydrates phases was determined at 2, 7 and 28 days of hydration (Figure 16). The mass loss between 50 °C and 140 °C is due to dehydration of the ettringite (E) and calcium silicate hydrates (C-S-H), as observed previously by [66], and increases with an increase in the hydration time for all the SCP specimens. The second mass loss is observed between 140 °C and 190 °C due to the dehydration of the monophases (AFm), similar to what was observed by [67], and is more pronounced in the SCP with RHA partial replacement in all hydration stages. Portlandite decomposition occurs between 400 °C and 450 °C for all SCP specimens, while calcium carbonate (CaCO_3_) decomposition takes place between 600 °C and 800 °C (Figure 16). The pattern of the formation of the hydrate phases is similar to what was observed previously by [54] when CC was used as a partial replacement for PLC in SCP.

The partial replacement of PLC by CC and RHA has a noticeable effect on the formation of hydrate phases, especially in the ranges of mass loss between 140 °C and 190 °C and between 400 °C and 450 °C. At 2 days of hydration, the DTA peak due to the dehydration of carbonate AFm phases is not evident in the SC-P and SC-P-20CC systems by TG measurements due to the increase of the Vw/Vp ratio used in this study, as the previous study by [54] noticed the formation of these phases in SC-P and SC-P-CC systems at the same time of hydration using a V_w_/V_p_ = 0.875. The reaction between the LP and CC enhanced the formation of the carbonate AFm phases at 7 and 28 days of hydration. This increased the volume of the hydration products in the SCP-CC system and densified its microstructure, as previously reported by [54]. RHA and the ternary blend of CC and RHA enhanced the precipitation of these phases even at 2 days of hydration and continued to increase up to 28 days of hydration.

**Figure 17:**
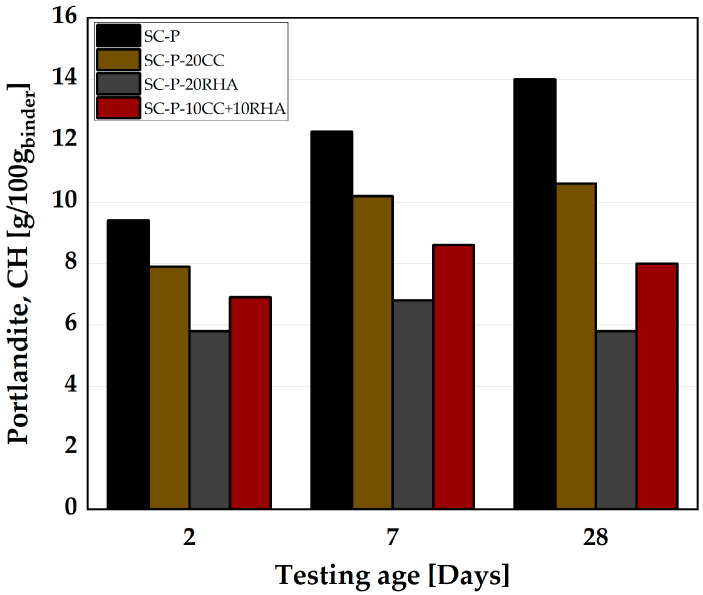
Influence of CC and RHA on portlandite (CH) consumption.

Figure 17 shows the influence of CC and RHA on portlandite consumption. SC-P has the highest CH content at each test time, while SC-P-20RHA exhibits the lowest CH content. SC-P had a CH content of 9.4 g at 2 days, 12.3 g at 7 days and 14 g at 28 days. The CH content of SC-P-20CC at 2 days was 7.9 g and increased to 10.2 g and 10.6 g at 7 and 28 days, respectively. SC-P-20RHA had a CH content of 5.8 g at 2 days, which increased to 6.8 g at 7 days and decreased to 5.8 g at 28 days. The CH content of SC-P-10CC+10RHA is halfway between that of SC-P-20CC and SC-P-20RHA at all test times.

The CH content of SC-P increases over the experimental period due to the continued hydration of C_3_S and C_2_S in the clinker portion of PLC. The use of CC and RHA as SCM reduced at 2 days of hydration the CH content, mainly due to the dilution effect. Simultaneously, CC and RHA provide more nucleation sites for the precipitation of hydration products at this stage of hydration, as previously observed by [54,68]. The relative decrease in CH content at 7 days of hydration is due to the dilution effect and the initiation of the pozzolanic reaction of CC and RHA, which consumes CH. At 28 days of hydration, CH consumption is significant, especially in SCPs with RHA as a partial substitute for PLC. This is indeed expected and attributed to the pozzolanic effect of CC and RHA, which consumed the CH and produced more C-S-H, which densified the SCP microstructure and thus increased the strength of the SCC, as can be seen later in Section 4.5.2.

### 4.5. Plastic and Hardened Properties of Self-Compacting Mortar and Concrete

#### 4.5.1. Effect of CC and RHA on Plastic and Total Shrinkage of Self-Compacting Mortar

**Figure 18:**
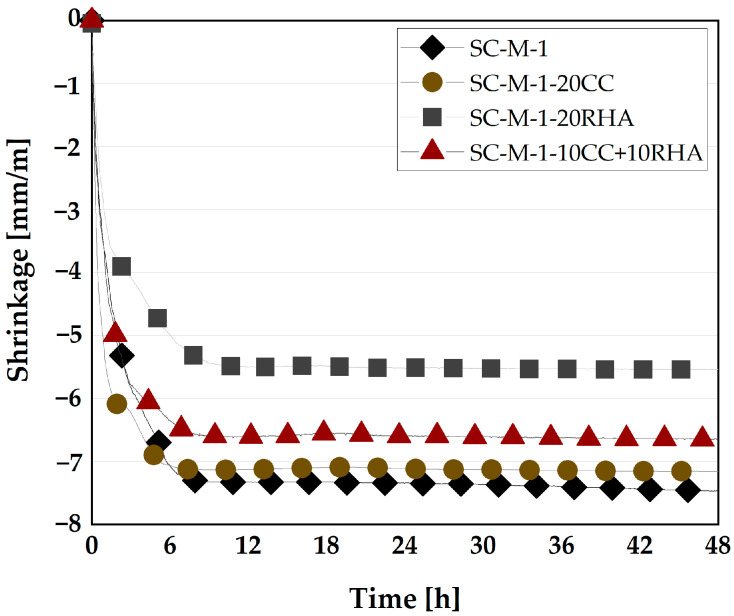
Effect of CC and RHA on plastic shrinkage of SC-M.

The mechanism of SC-M shrinkage during early hydration can be explained in three stages. First, plastic shrinkage, during the very early hydration phase, from water addition to about 7 h. The SC-M experiences a transition from fluid to a plastic material. During this period, the rate of plastic shrinkage of SC-M-1 and SC-M-1-20CC is high, reaching up to −7.0 mm/m (Figure 18). This value is somewhat lower to −5.2 and −6.5 mm/m in SC-M-1-20RHA and SC-M-1-10CC+10RHA, respectively. This high plastic shrinkage value of SC-M at this stage is due to the particles settlement caused by gravity leading to an increased packing density of the SC-M and forcing the free water to rise to the surface of the SC-M, resulting in bleeding, as previously explained by [69], and evaporation of moisture from the surfaces of the SC-M leading to the formation of water menisci, which eventually create a negative capillary pressure that contracts the SC-M particles and consequently causes volumetric contraction, similar to was reported by [70,71]. It has been reported that the use of shrinkage-reducing admixtures (SRA) reduced the plastic shrinkage of SCC at this stage of hydration by reducing the internal friction angle between SCC particles due to their high fluidity and delaying the setting time of SCC [69]. RHA is expected to behave similarly to the SRA in reducing the plastic shrinkage of the SC-M because it delays the initial setting time of the blended cement [72]. The second stage lasts from 7 to 16 h after water addition. Here, the plastic shrinkage rate of SC-M-1 decreases and yields a shrinkage value of −7.3 mm/m, while SC-M-20CC exhibits no further plastic shrinkage. Plastic shrinkage increases for the two other SC-M yielding −5.5 mm/m for SC-M-20RHA and −6.6 mm/m for SC-M-10CC+10RHA. Finally, after 16 h of hydration, the plastic shrinkage remains constant until the end of the measurement. The reason for the plastic shrinkage of the SC-M beyond 7 h of hydration could be due to the continuous evaporation of moisture from the SC-M surfaces and volume reduction due to water consumption by the hydration process, as previously observed by [54,70,71]. In general, the reduction in plastic shrinkage after 7 h of hydration due to the partial replacement of CC and RHA in SC-M could be attributed to the fact that both act as nuclei for the formation of hydration products at an early time of hydration, thereby increasing the volume of hydration product and densifying the SC-M microstructure, as observed previously by [73] when studying the dominant factors on the early hydration of metakaolin-cement paste. And for the RHA and ternary blend of CC and RHA, the reduction in plastic shrinkage could also be due to the release of the early absorbed water by the RHA particles, thereby increasing the internal relative humidity of the SC-M and reducing the autogenous shrinkage at an early time of the SC-M, as RHA was used previously by [74] to mitigate the autogenous shrinkage of cement paste.

**Figure 19:**
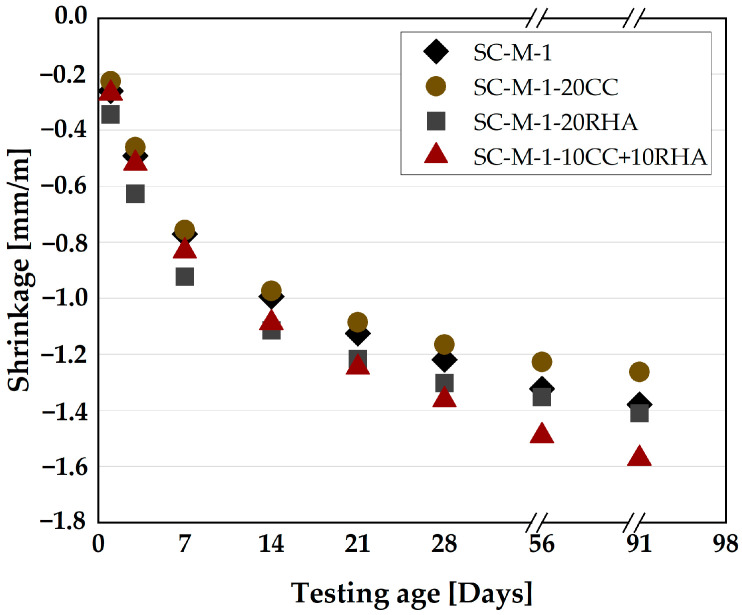
Effect of CC and RHA on drying shrinkage of SC-M.

The effect of the partial replacement of PLC with CC and RHA on the total shrinkage of SC-M was measured on 40 × 40 × 160 mm^3^ prisms, as presented in Figure 19. SC-M-1 and SC-M-1-20CC exhibited the same drying shrinkage values at all test times up to 14 days, after which SC-M-1-20CC shrank less than SC-M-1. SC-M-1-20RHA had the highest initial drying shrinkage of all SC-M specimens until about 14 days, when it decreased, yielding shrinkage values similar to SC-M-1 at 56 days. From 21 days and beyond, SC-M-1-10CC+10RHA shrank more than the other specimens.

The total shrinkage of SC-M specimens is a combination of drying shrinkage caused by the continuous loss of water from the capillary pores of the SC-M to equilibrate the relative humidity of the surrounding environment and autogenous shrinkage due to the reduction of water by the hydration process. SC-M-1 and SC-M-1-20CC have the same total shrinkage tendency up to 28 days of testing; beyond 28 days of testing, CC decreased the total shrinkage of the SC-M partly due to the volume increase of hydration product from the formation of more carboaluminate AFm phases, as shown previously in Figure 16, and or due to lower amount of evaporable water because of the pozzolanic reaction of the CC, which consumes more water, as previously reported by [75] in the case of using metakaolin as partial replacement for cement. RHA and the ternary blend of CC and RHA in SC-M specimens exhibit a different pattern of total shrinkage. RHA-SC-M specimens shrank more than other specimens up to 7 days of testing due to rapid evaporation of the absorbed water from the surfaces of the RHA-SC-M specimens, as the absorbed water was reported by [76] as having higher mobility than the water in the capillary pores and could evaporate easily, and thus an increase of the drying shrinkage. Beyond 7 days of testing, the total shrinkage of RHA-SC-M decreased and was equal to that of PLC-SC-M from 56 days and 91 days. The improvement of the total shrinkage could be due to the further release of absorbed water by RHA to restrain the decrease of the internal relative humidity of SC-M and, thus, a decrease in the total shrinkage. This phenomenon was explained previously by [74] when RHA was used as an internal curing agent in cement paste. From 21 days of testing and beyond, SC-M-1-10CC+10RHA shrank more than any other specimen due to continuous evaporation of the absorbed water from its surfaces.

#### 4.5.2. Compressive Strength and Rapid Chloride Migration Assessment of SCC

**Figure 20:**
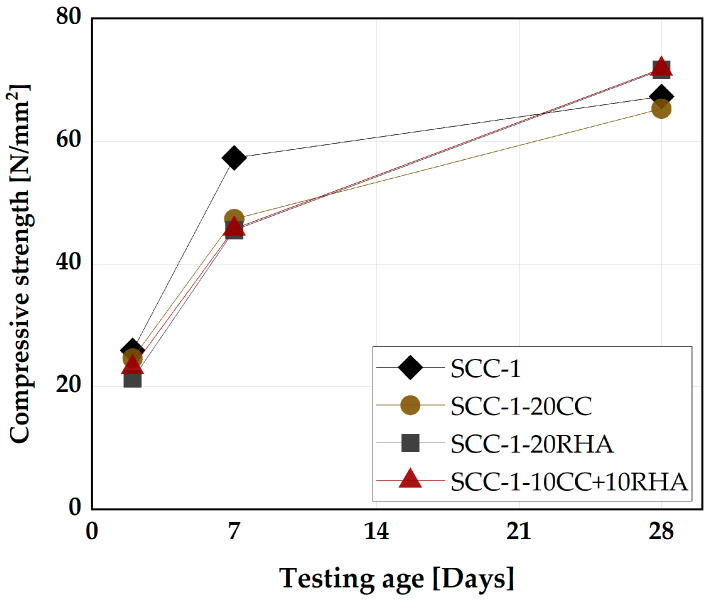
Compressive strength development of SCC specimens.

The impact of the CC and RHA on the development of compressive strength of SCC up to 28 days is shown in Figure 20. SCC-1 achieved higher values of compressive strength at 2 and 7 days. All the remaining specimens exhibited similar compressive strength values at 2 and 7 days, which are 8% and 21% lower than SCC-1, respectively. At this stage of curing, CC and RHA behaved similarly, and their physical presence resulted in a dilution effect and, thus, a reduction of the compressive strength, as observed previously by [54] when CC was used as a partial replacement for PLC in SC-M. At 28 days, SCC-1 and SCC-1-20CC reach similar compressive strength values while a slight increase of 6% is observed compared to SCC-1 for SCC-1-20RHA and SCC-1-10CC+10RHA due to the pozzolanic reactive of CC and RHA. The use of RHA and the blend of CC and RHA at 28 days of curing behaved similarly to silica fume in improving the compressive strength of SCC beyond the level of the pure cement, as previously observed by [77,78]. Another explanation for the increase of the compressive strength of RHA and the ternary blend of CC and RHA specimens is the continuous transition of Ca^2+^ from the PLC matrix to RHA, which enhances the pozzolanic reactivity of the RHA blended SCC and improves its compressive strength as explained by [79].

### 4.6. Rapid Chloride Resistance of SCC 

**Figure 21:**
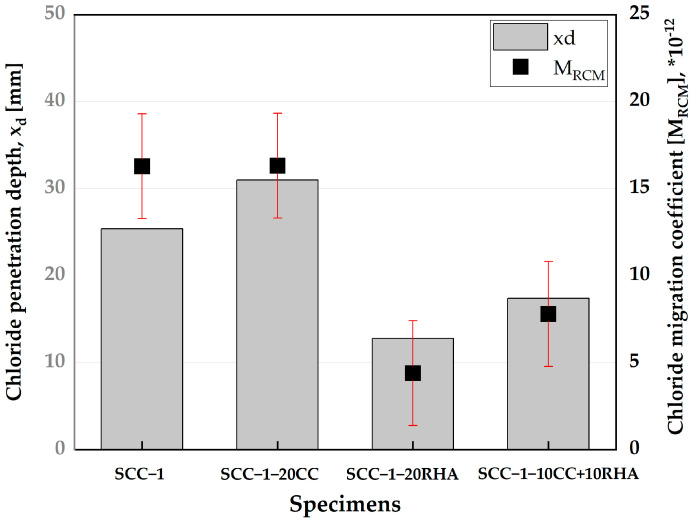
Influence of CC and RHA on the chloride resistance of SCC.

The depth of chloride penetration and migration coefficient of all SCC specimens are shown in Figure 21. The depth of chloride penetration of SCC-1 is 26 mm and increases to 30 mm for SCC-1-20CC. SCC-1-20RHA had the lowest chloride penetration depth of 13 mm, while that of SCC-1-10CC+10RHA was 19 mm. There is no significant difference between the chloride migration coefficient of SCC-1 and SCC-1-20CC, 16 × 10^−12^. SCC-1-20RHA had a chloride migration coefficient of 4.5 × 10^−12^ and increased to 8.2 × 10^−12^ for SCC-1-10CC+10RHA.

SCC-1-20CC achieved similar performance to SCC-1 in terms of chloride migration resistance; although the chloride penetration depth of SCC-1-20CC is greater than that of SCC-1, the determination of chloride migration resistance according to [53] considers other factors, such as the duration of the test and the voltage used, which give an indication for the density of a concrete microstructure. Both SCC-1 and SCC-1-20CC can be classified as having “Normal” SCC quality—8–16 × 10^2^ m^2^/s—according to non-steady state chloride migration resistance concrete classification by [80,81]. SCC-1-20RHA and SCC-1-10CC+10RHA achieved a “Good” SCC quality due to the pozzolanic reactivity of RHA that consumed CH from PLC hydration and produced more C-S-H, which densified the concrete microstructure and improved its chloride resistance.

## 5. Summary and Conclusions

- The study investigated the potential use of up to 40 vol-% CC and RHA as a partial replacement for PLC in SCP. Partial replacement of PLC with CC, as used in this study, is possible up to 40 vol-% and can be achieved with the same V_w_/V_p_ used for the PLC system, with an increase in SP dosages. The use of RHA as a partial replacement for PLC, on the other hand, requires urgent adjustment of the V_w_/V_p_ even at a lower replacement level of 5 vol-% to achieve similar deformability to the PLC systems; SP adjustment alone cannot provide the required degree of deformability. Therefore, for the application in SCC, the partial replacement of PLC by RHA should be kept at 20 vol-%;
- By adjusting the SP dosages, self-compactability can be achieved with PLC partial replacement with 20 vol-%CC, 20 vol-% RHA and 10 vol-% CC + 10 vol-% RHA, using a V_w_/V_p_ = 1.275;
- The deformability and short-term segregation resistance of the binary and ternary mix design with 20 vol-% CC, 20 vol-% RHA and 10 vol-% CC + 10 vol-% RHA are within acceptable limits, and therefore, the binary and ternary blends of CC and RHA could use in practice up to 20 vol-% as PLC partial replacement;
- At an increased SP dosage, PLC partial replacement with 20 vol-% CC has less impact than the binary and ternary blend of 20 vol-% RHA and 10 vol-% CC + 10 vol-% RHA on flowability retention of SCC up to 60 min, and therefore, SCC-1-20CC can be used to produce both precast and ready-mix SCC. However, the use of 20 vol-% RHA and the ternary blend 10CC+10RHA developed high flow resistance and showed rapid loss of flowability after 30 min of mixing. Therefore, their flowability retention needs to be improved for applications beyond 30 min. SCC with high content of RHA, 20 vol-% and above, is recommended for the production of precast SCC elements only due to the short flowability retention window required by precast SCC compared to ready-mix SCC. Although additional treatment may be required to improve the early strength development of RHA SCC;
- SCC produced with RHA as PLC partial replacement showed higher flow resistance and viscosity and increased both the static and dynamic yield stress of SCC. This effect is reduced to some extent by ternary blending CC and RHA. Therefore, the proportion of RHA shall always be kept low, perhaps at 5 vol-%, in the binary and ternary blended SCC mixture, when flowability retention beyond 30 min is required, for example, in the ready-mix concrete;
- Partial replacement of PLC with 20 vol-% CC reduced the plastic shrinkage of SC-M by 4%, 20 vol-% RHA reduced the plastic shrinkage of SC-M by 26%, while the ternary blend of 10 vol-% CC+10 vol-% RHA reduced the plastic shrinkage by 11%. Partial replacement of PLC with 20 vol-% CC had no effect on the total shrinkage of SC-M at 28 days of curing, while the binary and ternary blends of RHA, CC and RHA increased the total shrinkage of SC-M by 8 and 16%, respectively. Therefore, RHA could be an effective SCM to reduce hairline cracking that occurs at an early time in concrete due to the use of a high amount of cement per m^3^ of concrete;
- Both CC and RHA consumed CH due to their pozzolanic reactivity. Partial replacement of 20 vol-% PLC with CC had no significant effect on the 28-day compressive strength and chloride migration resistance of SCC. While the SCC produced with RHA and the blend of CC and RHA increased the 28-day compressive strength of SCC by 5%. The chloride migration resistance of 20 vol-% RHA is 3 times that of SCC produced with only PLC, while that of the ternary blend 10 vol-% CC + 10 vol-% RHA is 2 times that of SCC produced with only PLC. RHA is capable of improving the chloride migration resistance of SCC and should be used to improve the microstructural densification of SCC produced with only PLC.

## Figures and Tables

**Table 1:** Mineralogical composition of OPC and LP.

Materials	C_3_S	C_2_S	C_3_A	C_4_AF	Calcite	Sulfates	Quartz
OPC	61.6	18.2	5.8	9.0	0.6	3.2	-
LP	-	-	-	-	99.8	-	0.2

**Table 2:** Physical properties of OPC, LP, CC and RHA.

Properties	Methodology	OPC	LP	CC	RHA
Specific surface area, m^2^/g	DIN ISO 9277 [38]	1.0	1.6	3.9	160
Water demand, wt.%	Puntke method [39]	29	20	38	96
Particle density, g/cm^3^	DIN EN ISO 17892-3 [40]	3.29	2.81	2.65	2.4
d_10_, µm	Bettersizer 3D instrument [26]	2.6	0.8	1.9	5.4
d_50_, µm		16.0	4.6	12.7	23.7
d_90_, µm		42.8	20.7	33.7	56.5

**Table 3:** Average V_w_/V_p_ required by individual binder system to achieve self-compactability.

	CC	0	5	10	15	20	25	30	35	40
RHA										
0		0.87		0.87		0.91		0.94		0.98
5			1.1		1.08		1.12		1.17	
10		1.10		1.16		1.2		1.24		
15			1.21		1.25		1.27			
20		1.26		1.3		1.35				
25			1.34		1.42					
30		1.37		1.47						
35			1.50							
40		1.51								

**Table 4:** SCP mix designation.

Mix Designation	Constituent (Measured in dm^3^/m^3^)						Constituent (Measured in kg/m^3^)						
	V_w_/V_p_	OPC	LP	CC	RHA	Water	w/b	OPC	LP	CC	RHA	Water	SP [wt-%]
SC-P	1.275	374	66	-	-	560	0.4	1231	185	-	-	560	0.05
SC-P-20CC	1.275	299	53	88	-	560	0.4	984	148	233	-	560	0.1
SC-P-20RHA	1.275	299	53	-	88	560	0.4	984	148	-	211	560	0.3
SC-P-10CC+10RHA	1.275	299	53	44	44	560	0.4	984	148	116	106	560	0.2

**Table 5:** SC-M mix designation.

Mix Designation	Constituent (Measured in dm^3^/m^3^)							Constituent (Measured in kg/m^3^)							
	V_w_/V_p_	OPC	LP	CC	RHA	Water	FA	w/b	OPC	LP	CC	RHA	FA	Water	SP [wt-%]
SC-M-1	1.275	224	40	-	-	336	400	0.4	738	111	-	-	1104	336	0.15
SC-M-1-20CC	1.275	179	32	53	-	336	400	0.4	591	89	140	-	1104	336	0.2
SC-M-1-20RHA	1.275	179	32	-	53	336	400	0.4	591	89	-	127	1104	336	0.4
SC-M-1-10CC+10RHA	1.275	179	32	26	26	336	400	0.4	591	89	70	63	1104	336	0.3

**Table 6:** SCC mix designation (dm^3^/m^3^).

Mix Designation	Constituent (Measured in dm^3^/m^3^)								
	V_w_/V_p_	OPC	LP	CC	RHA	Water	FA	CA	V_a_
SCC-1	1.275	137	24	-	-	206	289	323	20
SCC-1-20CC	1.275	110	19	32	-	206	289	323	20
SCC-1-20RHA	1.275	110	19	-	32	206	289	323	20
SCC-1-10CC+10RHA	1.275	110	19	16	16	206	289	323	20

**Table 7:** SCC mix designation (kg/m^3^).

Mix Designation	Constituent (Measured in kg/m^3^)								
	w/b	OPC	LP	CC	RHA	Water	FA	CA	SP [wt-%]
SCC-1	0.4	452	68	-	-	206	798	867	0.25
SCC-1-20CC	0.4	362	54	86	-	206	798	867	0.3
SCC-1-20RHA	0.4	362	54	-	78	206	798	867	0.7
SCC-1-10CC+10RHA	0.4	362	54	43	39	206	798	867	0.5

## Data Availability

Not applicable.
